# Supplementary material for: Distribution of Polysulfide in Human Biological Fluids and Their Association with Amylase and Sperm Activities
Source: Molecules. 2019 Apr 30;24(9):1689. doi: 10.3390/molecules24091689 (PMC6539915; doi:10.3390/molecules24091689)
Supplement: Supplementary file 1 [file molecules-24-01689-s001.pdf]

# Supporting Online Information for

Distribution of Polysulfide in Human Biological Fluids and Their Association with Amylase and Sperm Activities

Mayumi Ikeda, Yu Ishima, Victor T. G. Chuang, Maki Sakai, Hiroki Osafune, Hidenori Ando, Taro Shimizu, Keiichiro Okuhira, Hiroshi Watanabe, Toru Maruyama, Masaki Otagiri, Takaaki Akaike, Tatsuhiro Ishida

Correspondence to: ishima.yuu@tokushima-u.ac.jp

**This file includes:**

Supporting methods

**Methods**

Determination of thiol contents in plasma

Twenty microliter of plasma was mixed into 100 μL of 5 mM DTNB in 100 mM of potassium phosphate buffer/ 1 mM DTPA (pH 7.0). After incubation for 60 min at room temperature, absorbance at 412 nm was measured by a plate reader. GSH (31.3 to 1000 μM) was used for constructing a standard curve.

Measuring anti-oxidative activity against AAPH radical [1]

Sixteen millimolar of linoleic acid solution was prepared by mixing 5 mL of borate buffer (50 mM, pH 9.0), 250 μL of linoleic acid, 1 mL of sodium hydroxide and 250 mL of tween20 and diluting in measuring cylinder to 50 mL by borate buffer (50 mM, pH 9.0). AAPH was dissolved in cold water on ice. Nine hundred twenty microliter of phosphate buffer saline (PBS) preheated at 37 $^{o}$C and 20 μL of plasma were mixed and 10 μL of linoleic acid solution (16mM) was added. Fifty microliter of AAPH solution (50 mM) was added and incubated for 1 hr at 37$^{o}$C. After the reaction, the sample solution was dispensed into 96 well of ultraviolet plate and absorbance read at 234 nm. Radical elimination activity was calculated as follows:

Eliminated radical (%) = (Abs. at 234 nm of sample with AAPH - Abs. at 234 nm of sample without AAPH) x 100 / (Abs. at 234 nm of PBS with AAPH - Abs. at 234 nm of PBS without AAPH)

Detection of sulfane sulfur by a fluorescence probe [2]

Plasma was diluted in 1 mL of 1 mM CTAB/PBS. 2 mL of 1 mM SSP4 in DMSO was added and incubated for 10 min at room temperature. Fluorescence intensity was measured at ex/em = 457 nm/514 nm.

Statistical analysis

Differences between the groups were evaluated by Welch's t-test. $P < 0.05$ was regarded as statistically significant.

**References**

[1] Kontush, A.; Reich, A.; Baum, K.; Spranger, T.; Finckh, B.; Kohlschütter, A.; Beisiegel, U., Plasma ubiquinol-10 is decreased in patients with hyperlipidaemia. *Atherosclerosis* **1997,** 129, (1), 119-126.

[2] Chen, W.; Liu, C.; Peng, B.; Zhao, Y.; Pacheco, A.; Xian, M., New fluorescent probes for sulfane sulfurs and the application in bioimaging. *Chem Sci* **2013,** 4, (7), 2892-2896.
